# A six‐year review of more than 13,000 patient‐specific IMRT QA results from 13 different treatment sites

**DOI:** 10.1120/jacmp.v15i5.4935

**Published:** 2014-09-08

**Authors:** Kiley B. Pulliam, David Followill, Laurence Court, Lei Dong, Michael Gillin, Karl Prado, Stephen F. Kry

**Affiliations:** ^1^ The University of Texas Graduate School of Biomedical Sciences at Houston Houston TX; ^2^ Department of Radiation Physics The University of Texas MD Anderson Cancer Center Houston TX; ^3^ Department of Radiation Oncology Scripps Proton Therapy Center San Diego CA; ^4^ University of Maryland School of Medicine Baltimore MD USA

**Keywords:** IMRT QA, quality assurance, patient‐specific QA, gamma

## Abstract

Due to a lack of information regarding the current clinical experience of IMRT QA for a large and varied plan population, we reviewed our patient‐specific IMRT quality assurance (QA) results for 13,003 treatment plans from 13 distinct treatment sites from a six‐year period. QA records were reviewed for dose difference (single point with ion chamber measurement; ±3% agreement criteria) and percentage of pixels passing relative dose gamma analysis (film measurement; 90% passing 5%(global)/3 mm agreement criteria) from 2005 through 2011. Plan records were analyzed for trends with measurement date and treatment site. Plans failing to meet QA tolerance criteria were evaluated for follow‐up clinical action (i.e., if repeat measurements were performed). The mean difference (±SD) between ion chamber point measurements and calculated doses was ‐0.29% ±1.64% (calculated values being slightly higher) and, regarding planar dose evaluations, the mean percentage of pixels passing the gamma criteria of 5%(global)/3 mm was 97.7% (lower 95th percentile: 92.2%). 97.7% and 99.3% of plans passed the point dose and planar dose verification, respectively. We observed statistically significant differences (p<0.05) in both point dose and planar dose verification measurements as a function of treatment site (particularly for stereotactic spine and mesothelioma sites) and measurement date (average agreement improved with time). However, despite improved dosimetric agreement, the percentage of failing plans has remained nearly constant at 2.3%.

PACS numbers: 87.55.Qr, 87.55.km, 87.56.Fc

## I. INTRODUCTION

Patient‐specific quality assurance (QA) of intensity‐modulated radiation therapy (IMRT) plans is an important step in treatment plan verification. A wide range of techniques and measurement devices are employed for this QA, including ion chambers, detector arrays, film, and electronic portal imaging devices (EPIDs). Moreover, there is substantial variability in other aspects, such as acceptance criteria, analysis methods and metrics, and region of interest selection. A major challenge that this raises is that consistent action limits are not well established across these different platforms; it is the responsibility of individual institutions to establish their own QA acceptance criteria.

Although there is a noted trend in the current literature of QA moving towards new devices and metrics,^1^, ^2^, ^3^, ^4^, ^5^ many of these techniques and devices are still being verified clinically. Importantly, in some cases gamma evaluation fails to detect failing plans.^6^, ^7^ The historical standard for QA has been point dose evaluation and planar evaluation, and they have been viewed as a gold standard (per TG 119) for some time.^8^, ^9^, ^10^ Even though point dose and planar dose evaluations have been used for a long time, there still remains a large void in the literature about long‐term statistics on QA for a large population including a wide range of treatment sites.

Previous studies of clinical IMRT QA data^9^, ^10^ have found high pass rates and uniformity across treatment sites. However, none of the previous studies had a particularly large or varied population, limiting their ability to evaluate more challenging sites, such as mesothelioma. These studies also focused primarily on plans that passed QA tolerance criteria, and did not explore the distribution or characteristics of plans failing QA tolerance criteria, or follow‐up to such plans.

Therefore, in an effort to provide broad information on IMRT QA statistics, we retrospectively analyzed the point dose and planar dose evaluation results for more than 13,000 patient plans across 13 clinical treatment sites performed from 2005 through 2011 at The University of Texas MD Anderson Cancer Center (UT MDACC).

## II. MATERIALS AND METHODS

### A. QA program

Quality assurance records included 13,003 individual QA plans with 13,308 point dose and 12,677 relative planar dose measurements from January 2005 to July 2011. Patients with multiple plans and/or primary and boost plans were counted separately. These plans were distributed across 13 different treatment sites: breast (BRST), central nervous system (CNS), gastrointestinal (GI), genitourinary (GU), gynecology (GYN), hematology (HEM), head and neck (HN), intensity‐modulated stereotactic spine radiation therapy (IMSSRT), melanoma (MEL), mesothelioma (MESO), pediatric (PEDI), sarcoma (SAR), and thoracic (THOR). The number of plans on each site is shown in Table 1. The majority of IMRT treatments were planned using 6 MV X‐rays and some with 18 MV X‐rays. The treatments were delivered by Clinac 2100 or 600 linear accelerators (Varian Medical Systems, Palo Alto, CA). The IMRT technique was mainly step and shoot, although during the latter years there has been an increase of VMAT. The typical number of beams numbers depended on the treatment site, as shown in Table 1

**Table 1:** IMRT QA results by treatment site from 2005 to 2011

*Treatment Service*	*Typical Number of Beams*	*Number of Plans*	*Mean Ion Chamber Difference (%)*	*1 SD*	*Number of IC Failing Plans/(% of Failing on Service)*	*Mean Gamma (%)*	*Lower 95* ^*th*^ *percentile (%)*	*Number of Gamma Failures/(% of Failing on Service)*
BRST	Varies	67	0.08	1.61%	2 (3.0)	97.9	93.1	0 (0.0)
CNS	5	1383	‐0.23	1.43%	13 (0.9)	97.9	92.6	7 (0.6)
GI	6	803	0.45	1.80%	33 (4.1)	97.2	91.6	5 (0.6)
GU	8	1831	‐0.17	1.18%	11 (0.6)	97.6	92.5	2 (0.1)
GYN	9	935	0.29	1.61%	23 (2.5)	97.6	92.0	8 (0.9)
HEM	5‐7	380	‐0.16	1.66%	7 (1.8)	97.7	93.2	2 (0.5)
HN	7‐9	3697	‐0.45	1.62%	76 (2.1)	97.7	92.3	33 (0.9)
IMSSRT	9	341	‐1.59	2.79%	54 (15.8)	97.6	92.9	4 (1.2)
MEL	5‐7	54	‐0.04	1.66%	1 (1.9)	97.2	92.1	0 (0.0)
MESO	7^a^	52	2.60	2.58%	11 (21.2)	94.4	86.4	6 (11.5)
PEDI	Varies	307	‐0.25	2.01%	18 (5.9)	97.8	92.0	2 (0.7)
SAR	5‐7	201	0.12	1.50%	6 (3.0)	97.4	91.9	3 (1.5)
THOR	8	2952	‐0.53	1.44%	46 (1.6)	97.8	92.2	23 (0.8)
Totals	N/A	13,003	‐0.29	1.64%	301 (2.3)	97.7	92.2	95 (0.7)

a Although the number of beams is seven on average, each of these treatment beams may have up to three carriage positions to allow for treatment of the large fields associated with mesothelioma plans.

Pinnacle^3^ treatment planning system (TPS; Philips Healthcare, Fitchburg, WI) was used for planning. A pretreatment verification plan was prepared by recalculating dose distributions on a homogenous IMRT QA phantom. During the six years evaluated in this study, the Pinnacle TPS version changed as follows: version 6 was used until early 2005, version 7 from early 2005 to mid‐2008, version 8 from mid‐2008 to late 2010, and version 9 from late 2010 to present.

**Figure 1:**
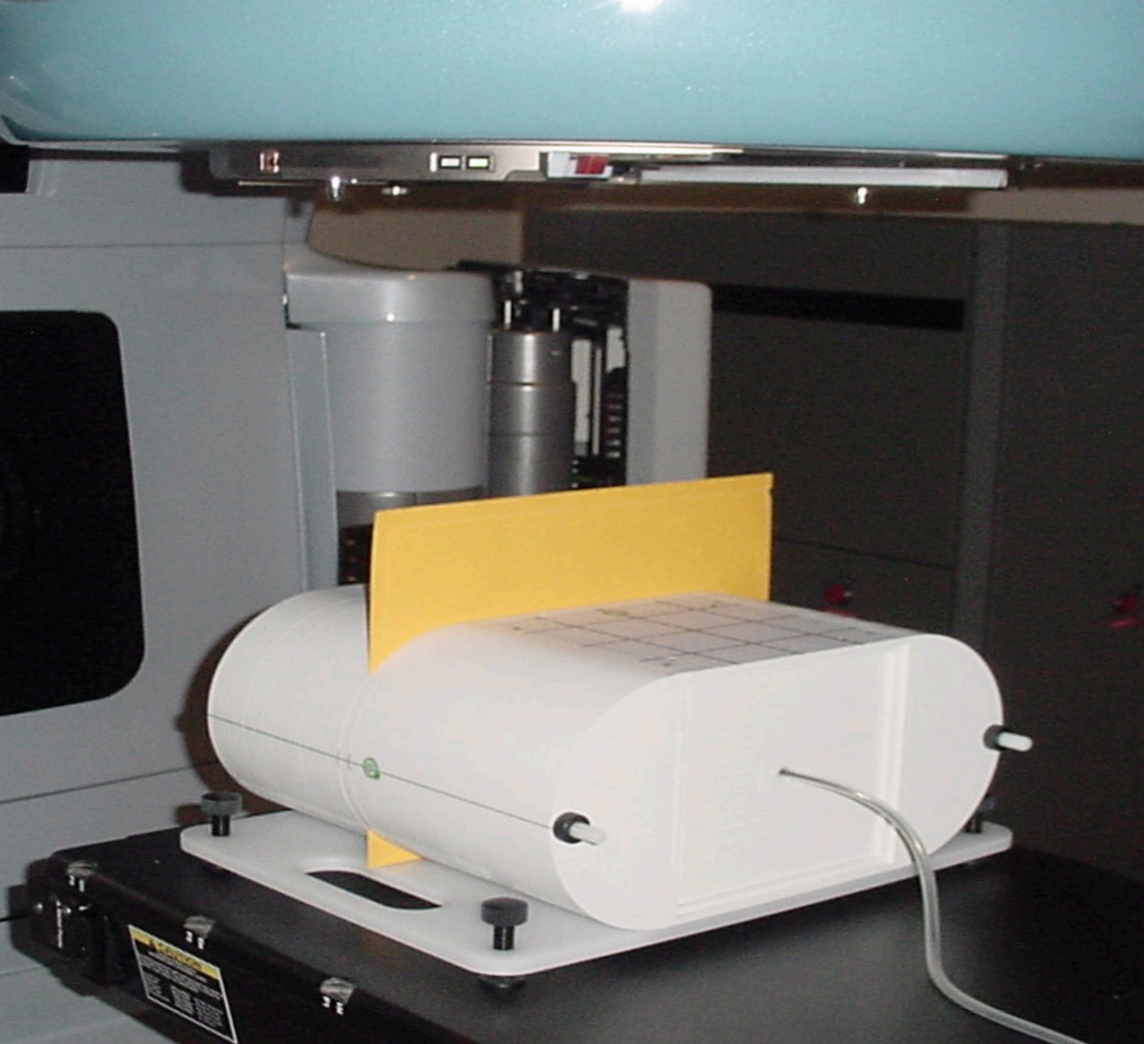
IMRT QA set‐up used at MD Anderson starting in August 2005. A CC04 ion chamber and Extended Dose Range 2 Ready Pack are inserted for measurement.

Pretreatment verifications consisted of point dose measurements in a high‐dose, low‐gradient region and planar relative dose measurements. For point measurements, an ion chamber traceable to a secondary calibration laboratory was used. The ion chamber used for point dose measurements was a Wellhofer model CC04 (0.04 cm^3^ active volume) ionization chamber (IBA Dosimetry, Bartlett, TN) corrected for temperature, pressure, and daily machine output variations, along with a Model 206 electrometers (CNMC, Nashville, TN). The ion chamber was placed in the center of the IMRT phantom and the ion chamber measurement compared to the TPS‐calculated dose over a region of interest corresponding to the ion chamber active volume. The ion chamber was used inside one of two phantoms. The majority (93%) of QA measurements were made in a water‐equivalent I'm*RT* Phantom (IBA Dosimetry) (Fig. 1; RW3 material 1.045g/cm^3^ density), whereas early measurements (through November of 2005) used a custom‐built, water‐filled phantom. For both phantoms, the QA measurements were performed at the treatment plan gantry angles. For the 2D measurements, Kodak Extended Dose Range 2 Ready Pack Film (Eastman Kodak, Rochester, NY) was used. The relative planar dose assessment was performed by measuring the dose in the transverse plane 2 cm superior to the ion chamber position in the I'm*RT* phantom with beams delivered at the planned gantry angles. The film was calibrated using eight dose levels, ranging from 77 to 587 cGy, to generate a calibration curve for conversion of optical density to dose. Films were developed using a Kodak RP X‐OMAT Processor (Eastman Kodak) and were digitized using the VXR‐16 Dosimetry Pro or Dosimetry Pro Advantage (VIDAR Systems Corporation, Herndon, VA). Each film measurement was digitized and then normalized to the point of maximum intensity for gamma calculations using the OmniPro‐I'm*RT* software (IBA Dosimetry). Two‐dimensional gamma analysis with OmniPro‐I'm*RT* did not begin until late 2005 as the original in‐house phantom did not include film measurements. Treatment plans passed QA if the measured‐to‐calculated ion chamber dose agreed within ±3%, and at least 90% of the pixels passed gamma criteria of 5%(global)/3 mm with the measured film distribution used as the evaluated distribution. These gamma calculations were performed using no additional processing such as dose thresholding, dose grid sampling, or interpolation.

Analyzed data included the majority of QA point dose measurements made from 2005 to 2011; in clinical practice QA point dose results slightly outside the ±3% point dose tolerance (e.g., 3.1%–3.2%) were typically remeasured immediately at the same location. If the second reading was within tolerance (e.g., 2.9%), only the final passing measurement was recorded. This process preserved the number of QA plans in the dataset, but underrepresented failed point dose measurements on the edge of acceptability.

### B. Review of QA results

QA records for each plan included date of measurement, clinical treatment site, measured and calculated dose, and percentage of pixels passing gamma. Duplicate measurements or test cases (for new software or equipment) were excluded. Additionally, to the extent possible, plans with measurement errors (e.g., phantom positioning errors) were excluded.

SPSS (IBM Corporation, Armonk, NY) was used to perform one‐way analysis of variance (ANOVA) with Tukey's honestly significant difference (HSD) test and the nonparametric Kruskal‐Wallis test on the data to evaluate significant differences as a function of treatment site and date.

### C. Failure of QA measurements

Additional analysis was conducted for plans that failed point dose and/or planar evaluation (referred to as plan failures), categorizing plans by follow‐up QA action and its success. Follow‐up action could include repeating the measurement at a different point (for point dose measurements only), remeasuring using a special beam‐delivery technique, documenting the failure and treating, scaling the monitor units (MUs) based on the QA results to split the difference between measured and calculated dose, replanning, or a combination of these techniques. Special beam‐delivery techniques included delivery of all beams at a 0° gantry angle (Anterior– Posterior (AP) beams) and, in plans with multiple carriage positions (i.e., large field plans, such as MESO, in which the MLC bank has to move to multiple locations at a single gantry angle, thereby delivering two or even three fields at a single gantry angle), separate assessment of the point dose measurements from each carriage position (Multicarriage (MC) split).

## III. RESULTS

### A. Point dose measurement differences

The mean dose difference between the measured and calculated ion chamber doses (±SD) was ‐0.29% ±1.64% (with the calculated dose slightly higher) (Table 1). This table also shows that, among the treatment sites, plans in IMSSRT and MESO had the largest mean dose differences (‐1.59% and 2.60%, respectively) and standard deviations (2.79% and 2.58%, respectively). The remaining 11 treatment sites had mean differences less than 1% and standard deviations 2% or less. Table 1 also shows the number of plans with percent differences larger than 3% and the percentage of these plans by site. Overall, 301 plans (2.3% of plans) had at least one failing point dose measurement. In addition to having the largest mean dose differences, IMSSRT and MESO had the highest rates of plan failure (16.1% and 21.2%, respectively), whereas failure rates for the remaining sites were 5.2% or lower.

**Figure 2:**
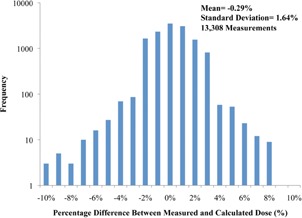
Point dose percent differences (measured versus calculated) for all measurements according to frequency on a logarithmic scale (six measurements outside the ±10% range are not shown).

Figure 2 shows the mean point dose difference results on a logarithmic scale binned by the recorded percent dose difference for all 13,308 recorded measurements. The majority of measurements were within ±3% tolerance, and the majority of failed plans (2.3%) were only a few percent outside tolerance. However, some measurements showed substantial disagreements.

Statistical differences in mean point dose differences between treatment sites (using a one‐way ANOVA with Tukey's HSD test) showed that, while the majority of treatment sites were statistically homogenous with numerous other sites, the mean point dose differences in the IMSSRT and MESO sites were statistically distinct from those in all other treatment sites (p<0.05).

**Figure 3:**
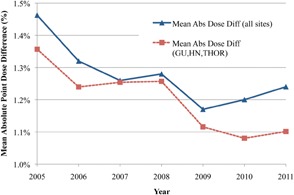
Difference in mean point dose differences by year for all treatment sites versus the three largest sites (HN, GU, and THOR) with connecting lines used for clarity.

**Figure 4:**
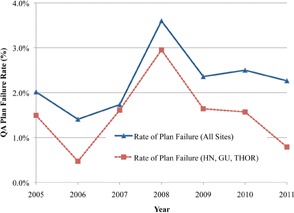
Point dose plan failure rates per year for the two groups in Fig. 3.

The mean dose differences per year for all treatment sites, as well as for the three largest treatment sites (GU, HN, and THOR), are shown in Fig. 3. The three largest treatment sites are plotted separately to remove any biasing effects for the smaller, less homogenous sites, such as IMSSRT and MESO. Figure 3 shows that the mean dose difference tended to decrease steadily for both groups (i.e., increased dosimetric agreement) from 2005 (1.46%) to 2011 (1.23%). The dosimetric agreement was better in GU, HN, and THOR subgroups than in the overall group, but the trends of agreement between both groups were consistent. There was also a significant (p<0.05; per ANOVA) change in the mean dose difference as a function of treatment year. Figure 4 shows the rates of point dose plan failure over all treatment sites and for the GU, HN, and THOR subgroup. Surprisingly, although the mean dose difference improved over time, the failure rates were essentially constant with time for both groups. The “all treatment sites” group had a higher rate of failure (2.3%) than the GU, HN, and THOR subgroup (1.5%) due to the inclusion of IMSSRT and MESO.

### B. Planar dose comparisons — gamma analysis

Gamma evaluations are shown in the last three columns of Table 1 for the 12,677 planar dose verifications. The overall mean percentage of pixels passing at 5%/3 mm was 97.7% (lower 95th percentile, 92.2%). As seen in Table 1, on average, the MESO plans had the poorest mean percentage of pixels passing (94.4%) and lower 95th percentile (86.4%). All other sites had mean passing percentages between 97% and 98%. Only 95 plans (0.7%) failed the gamma criteria, and almost all sites had failure rates less than 1%.

**Figure 5:**
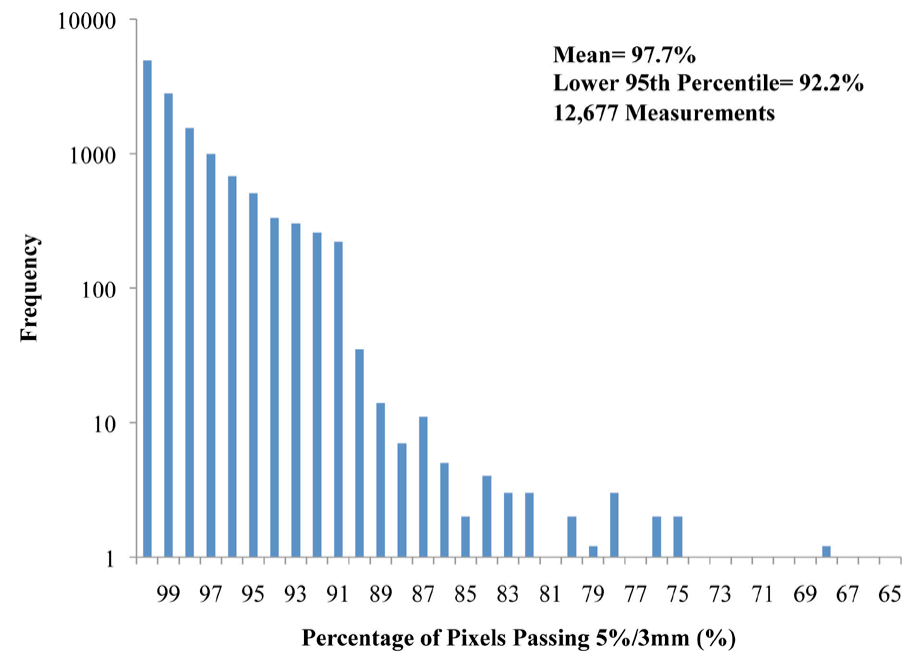
Percentage of pixels passing the 5%/3 mm gamma criteria for all gamma measurements according to frequency on a logarithmic scale. Bins with a frequency of 1 are shown as 1.2 for visualization purposes. (Four measurements with less than 65% of pixels passing are not shown.)

Gamma analysis results on a logarithmic scale (Fig. 5) show that the majority of failed measurements were within 10% of passing criteria (i.e., not less than 80% of pixels passing gamma), with only eight measurements outside this range (i.e., <80% of pixels passing 5%/3 mm).

**Figure 6:**
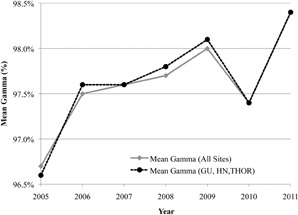
Mean percentage of pixels passing the 5%/3 mm gamma criteria by year for all treatment sites versus the three largest sites (HN, GU, and THOR).

**Figure 7:**
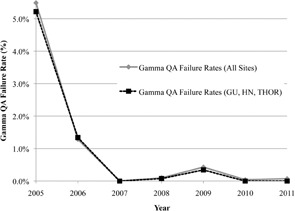
Gamma plan failure rates per year for the two groups in Fig. 6.

Gamma results were evaluated according to treatment site and year using the Kruskal‐Wallis test. The results showed a statistically significant (p<0.05) difference in gamma measurements as a function of both treatment site and year. MESO plans had the lowest mean rank. With respect to measurement year, 2005 and 2011 had the lowest and highest mean ranks, respectively. Mean percent of pixels passing gamma for all measurements and the GU, HN, and THOR subgroup are plotted in Fig. 6, showing a slight increase in mean gamma over time (approximately 2% change). The corresponding gamma failure rates are shown in Fig. 7, demonstrating a sharp decrease in gamma failures after 2006. Excluding 2005 and 2006, during which problems with the film processor caused the vast majority of the failing gammas, the mean gamma failure rate was only 0.1%.

### C. Failing QA plans

**Figure 8:**
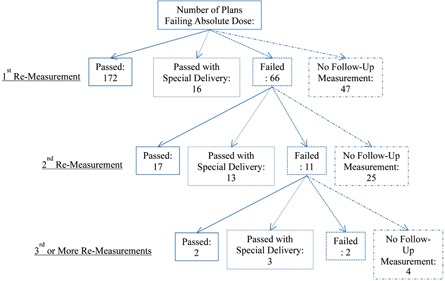
Flow chart showing the QA follow‐up performed on the 301 plans that failed point dose comparison.

Figure 8 shows a flow chart of the follow‐up for the 301 plans that failed point dose measurement QA. The majority of these plans passed (188) with a single additional measurement (at a new location), while 66 failed this remeasurement. With 2nd and 3rd or more remeasurement of failing plans, the portion of plans passing and failing remained fairly constant (average of 49% passing and 19% failing), but the portion of plans passing when using special delivery increased (from 9% to 60%). As plans continued to fail QA with normal delivery, the use of special delivery techniques (AP or MC split) increased, becoming the dominant manner that plans passed after three failing measurements. Of the 301 plans that failed initial point dose measurement, 191 eventually passed at a different location with normal irradiation conditions. In clinical practice, these plans were considered to have passed, and were treated on an as‐is basis. Eighteen plans passed with a special delivery technique; again, these plans were treated on an as‐is basis. A total of 76 plans had no recorded follow‐up passing measurement. Of these, 47 had no initial remeasurement; these plans were typically close to passing and clinical judgment was used to deliver these plans on an as‐is basis. Plans with no follow‐up after multiple failures typically reflected cases with known issues — MESO, IMSSRT, or large‐volume PEDI cases — where there were known limitations in the TPS (in modeling multiple carriage fields or very small fields). Of these plans, the vast majority were nevertheless treated on an as‐is basis following consultation with the radiation oncologist. A small portion of plans with large levels of disagreement (typically between 4% and 6%) had the plan MUs scaled up or down to bring the calculated dose into tolerance with the measured dose. These plans were then treated using the scaled MUs. Very infrequently, replanning was employed (only three cases noted). The exact course of corrective action also depended on the treatment site. For example, IMSSRT plans generally failed low (measured less than calculated dose); however, even for substantial dose disagreements, these plans were generally treated on an as‐is basis because of intolerance for overdosing the spine and the high likelihood that even as‐planned, some tumor would not be fully covered using IMRT. In summary, our general response to failing QA plans was to first to remeasure the plan at the same calculation point, then, if that failed again, to move to a new calculation point. If subsequent measurement(s) failed, then typically one of the following actions occurred: the failure was documented and the plan used as‐is, a special delivery technique was used to try to pass the plan, the plan MUs were scaled to split the difference with measurement, or the plan was modified.

For the 95 plans that failed to meet the gamma passing criterion, only four had follow‐up repeat measurements, all of which passed. The remaining 91 plans had no follow‐up measurements. All plans were treated on an as‐is basis. Of possible concern, no change to a clinical treatment plan ever occurred due to a plan failing gamma criterion.

In general, point dose and planar dose evaluation failures were only mildly associated with each other. Of the 95 gamma‐failed plans, only five had corresponding point dose failures. Poorer point dose agreement corresponded to lower, but rarely failing, gamma measurements.

## IV. DISCUSSION

Our review of 13,003 patient‐specific IMRT QA plans highlights a number of major and minor issues. First, the proportion of IMRT QA point dose failures was not negligible; 2.3% of all plans failed the point dose measurement, including 66 plans (22% of the failures) that failed at two or more measurement locations. Second, improved dosimetric agreement between TPS and measured dose did not reduce the rate of QA plan failure — while point dose agreement improved over time, plan failure rates remained relatively constant. Third, our planar analysis techniques detected dosimetric plan errors with a much lower frequency than our point dose measurements (<0.1% of plans during the past five years for film versus 2.3% of plans for the ion chamber).

Our passing rates for point dose and planar dose (97.7% and 99.3%, respectively) are consistent with prior studies: 97%–99% point dose agreement^8^, ^9^ and 97.7% planar dose agreement (gamma analysis)^(9)^ However, in contrast with previous findings, our results were statistically different among treatment sites. This difference is attributable to our study's increased number and variety of treatment sites, especially our inclusion of the high‐failing MESO and IMSSRT sites.

Although we observed a roughly constant point dose plan failure rate, we observed a steady improvement in dosimetric agreement between TPS and measured dose over the years (Fig. 3). The periods of improvement (2005‐2006 and 2008‐2009) roughly coincided with the clinical implementation of new versions of the Pinnacle TPS, which often included improved optimization algorithms. However, the general clinical practice in our institution of using less modulation (fewer MLC segments and MUs) also may have contributed to the improved agreement. Additionally, systematic errors, such as those inherent to beam modeling, and the impact of beam attenuation by couch structures^(11)^ (which were not accounted for, although the rails were always moved out of the way of the beams) could also be contributing factors.

Among the point dose failures (Fig. 5), the majority (74%) of plans ultimately passed using one or more additional calculation point measurements. A subset of these plans that ultimately passed (11%) did so only after use of a special beam delivery technique (AP or MC split). These delivery methods are considered secondary techniques because neither represents how a plan will be delivered clinically. Our results suggest that the two special beam delivery approaches may be more forgiving in enabling plans to pass IMRT QA, as plans that failed almost always subsequently passed using special delivery techniques (although not universally, as five plans failed a special delivery). This finding is especially important as many institutions exclusively use an AP delivery for IMRT QA with their measurement devices. The use of special beam delivery approaches, along with scaling of MUs, is not a resolution to IMRT QA failures that is necessarily endorsed by this work. Indeed, the entire process of repeating measurements until an acceptable result is obtained should be viewed with alarm by the medical physics community. More satisfying options to resolve plans that fail IMRT QA should be desired. However, particularly given the practical challenges of clinical workflow, this issue is not trivial.

Unlike our point dose failures, the rate of planar failure decreased over time (Fig. 7). More than half of the gamma failures occurred in 2005 and 2006 and were attributed to problems with the film processor. Without processor errors, the gamma failure rate was around 0.1% (Fig. 7). Unfortunately, our planar QA was, therefore, more sensitive to dosimeter/processor problems than to treatment plan problems; moreover, even when the planar analysis failed, no treatment plans were ever modified because none of the failures was interpreted as clinically serious by the radiation oncologists. A relatively low sensitivity of planar analysis to detect plan errors has also been noted in the literature for AP irradiations^6^, ^7^. For our QA, all beams were delivered at the planned angles. However, other issues may have contributed to the much lower error rate than was observed with the point measurements made with an ion chamber. Specifically, our gamma criteria may be too lax, and no dose thresholding was employed. To increase the sensitivity of our planar analysis, more stringent percent of pixels passing or dose difference/DTA could be employed (for example, 3%/3 mm, which is commonly used with planar devices)^(12)^. For example, to generate 2.3% of plans failing gamma (thereby matching the of point dose failure rate), the percentage of pixels passing 5%/3 mm would have to be increased from 90% to 96.6%. It is not clear that such a high threshold for percent of pixels passing is reasonable.

Ideally, all QA devices would be (at least approximately) equally able to detect unacceptable plans. Because the goal of QA is to detect plans that contain dosimetric errors and deviations from what is expected, all devices should have the same passing rates. However, in reality, this is not the case. The study by McKenzie, et al.^(13)^ showed that for a set of acceptable and unacceptable plans, the sensitivity and specificity in correctly labeling these plans varied between different detectors (MapCHECK, ArcCHECK, film, and single ion chamber). The physics community should work to at least standardize the results of different IMRT QA devices so that more consistent results can be achieved. In that sense, the present review serves as a reference point for the QA program used at our hospital. The failure rate shown will not necessarily apply to all hospitals, but it provides a basis for further comparison. Future work is needed to define relevant action levels and expected failure rates for new QA devices, similar to the work of Howell et al^(14)^. Additional work is needed to define action limits based on clinically meaningful outcomes because the current limits are typically arbitrary.

## V. CONCLUSIONS

Our review of 13,003 patient‐specific IMRT QA plans found a large difference in sensitivity to dosimetric errors between point dose and planar gamma analysis, specifically that using our gamma criteria did not result in appreciable plan error detection. Additionally, we found that, despite improvement in dosimetric agreement over time, plan failure rates remained nearly constant and at a nontrivial rate (2.3%); therefore, QA programs with no plan failures may have QA techniques and/or action levels that are not sensitive to plan errors. We also found that there were significant and substantial differences in the QA agreement between different treatment sites.

## ACKNOWLEDGMENTS

The authors thank physics assistants Scott LaNeave, Craig Martin, Jared Ohrt, Andrea Ohrt, Luke Whittlesey, Miguel Herrera, and Nicholas Murray for maintaining the QA records. This research is supported in part by the National Institutes of Health through RPC grant CA010953 and MD Anderson's Cancer Center Support Grant CA016672.
